# Controlled Periodic Illumination Enhances Hydrogen Production by over 50% on Pt/TiO_2_

**DOI:** 10.1021/acscatal.1c01734

**Published:** 2021-05-18

**Authors:** F. Sordello, F. Pellegrino, M. Prozzi, C. Minero, V. Maurino

**Affiliations:** † Dipartimento di Chimica and NIS Center, University of Torino, Via P. Giuria 7, 10125 Torino, Italy; ‡ JointLAB UniTo-ITT Automotive, Via Quarello 15/A, 10135 Torino, Italy

**Keywords:** controlled periodic illumination, hydrogen evolution reaction, titanium dioxide, photoreforming, volcano plot, sabatier, surface catalytic resonance

## Abstract

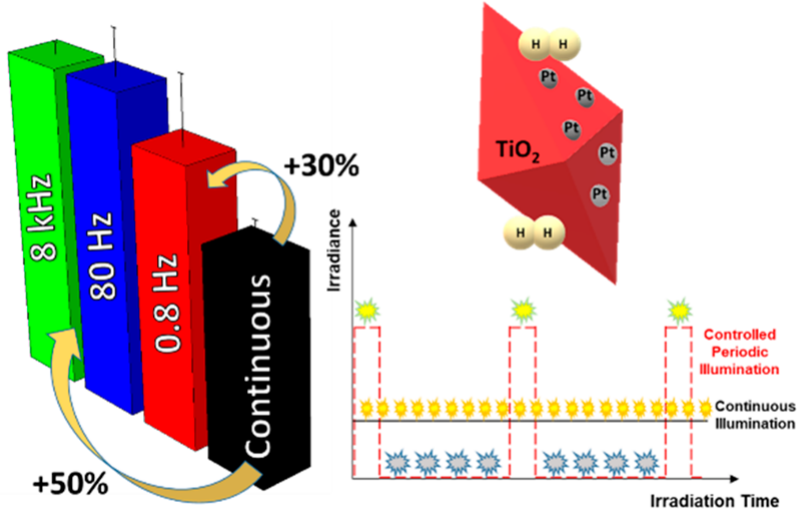

Efficient solar water photosplitting is plagued by large overpotentials of the HER and OER. Even with a noble metal catalyst, the hydrogen evolution reaction can be limited by the strong M–H bonding over some metals, such as Pt, Pd, and Rh, inhibiting hydrogen desorption. H absorption is regulated by the potential at the metal nanoparticles. Through controlled periodic illumination of a Pt/TiO_2_ suspension, we hypothesized a fast variation of the photopotential that induced catalytic surface resonance on the metal, resulting in more than a 50% increase of the efficiency at frequencies higher than 80 Hz.

In the next decades, the conversion of solar energy into electricity and solar fuels will be of crucial importance for a green and sustainable future.^1^ However, many challenges remain to exploit solar energy in an efficient way.^2,3^ In this context, water splitting using semiconductor photocatalysts has been considered a sustainable method to produce clean hydrogen (H_2_) fuel.^4,5^ Nevertheless, H_2_ photoproduction efficiency still remains low, although extensive research effort has been carried out in recent years about the mechanisms of the Hydrogen Evolution Reaction (HER) and the Oxygen Evolution Reaction (OER).^6−8^ In this respect, TiO_2_ is a key photoactive material, usually employed with a cocatalyst deposited onto the surface to enhance charge carriers’ separation and catalyze surface charge transfer reactions.^9,10^

Among various cocatalysts, Pt often exhibits the best performances, placing it on the top of the Sabatier’s volcano plot. In accordance with the Sabatier principle, in a two-step reaction like H^+^ reduction, the interactions between the catalyst and the substrate should be optimal, neither too weak nor too strong. In the first case, the substrate adsorption at the metal surface will be poor, slowing the overall reaction. On the other hand, with a too strong interaction, the product dissociation fails.^11^ The rate of both steps depends on the local electrical potential on the Pt nanoparticles during illumination.

Irradiated slurries of Pt-loaded TiO_2_ can evolve H_2_ through photoreforming of organic compounds.^12−14^ Under irradiation, the Fermi level for electrons in TiO_2_ becomes sufficiently negative to trigger H_2_ evolution on catalytically active Pt islands deposited on the surface. Therefore, the deposition of a cocatalyst on the TiO_2_ surface represents a way to enhance the activity of the photocatalyst through a modification of its surface and redox properties.^14,15^ Another investigated strategy in the field of TiO_2_ photocatalysis to enhance quantum yield (or photonic efficiency) of the photocatalytic process consists of employing a temporal modulation of the light source, i.e., Controlled Periodic Illumination (CPI).^16,17^ In this technique, the time profile of the irradiance incident on the reaction cell consists of a light pulse characterized by (*i*) the peak irradiance (*I*^CPI^), (*ii*) the period (*P*) equal to the sum of light (*t*_ON_) and dark (*t*_OFF_) time, and (*iii*) the duty cycle (γ = *t*_ON_/*P*; see Figure 1).

**Figure 1:**
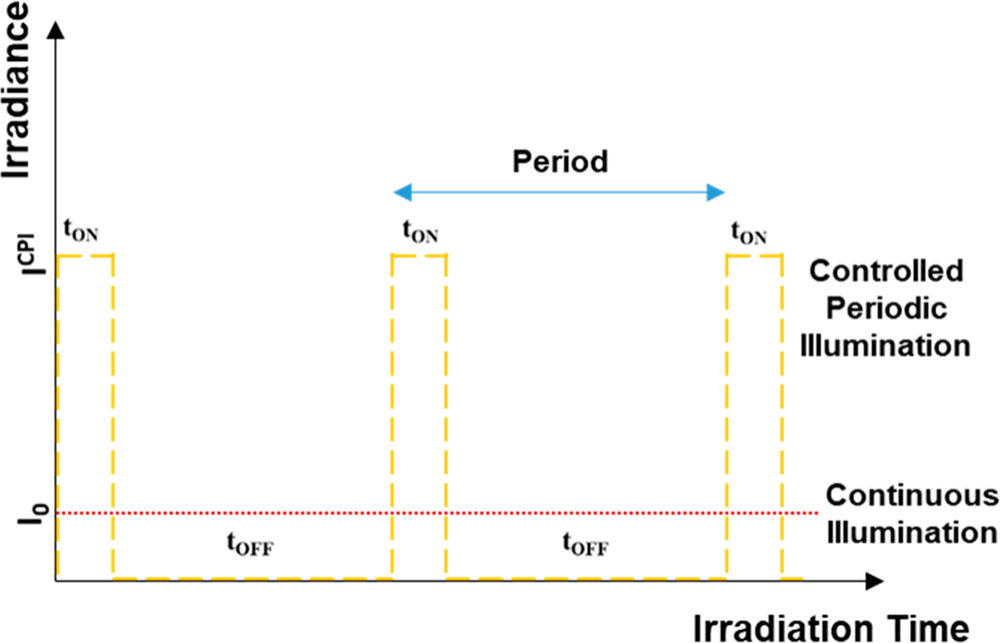
Incident irradiance vs time during a CPI experiment. I^CPI^ is the incident irradiance during the light time (t_ON_) of the CPI experiment (yellow dashed line), while t_OFF_ is the dark time. The red dashed line represents the constant irradiance incident on the reaction cell during a continuous illumination experiment (I_0_) having the same average irradiance of CPI.

Our research group recently published a paper in which it demonstrated that CPI is unable to increase the quantum yield of the photocatalytic process for pollutant abatement on a bare TiO_2_ suspension (oxidation of formic acid by P25 aqueous slurry).^18^ This result is coherent with the previous observations of Cornu et al.^19,20^ and with the theory of intermittent illumination developed by Melville and Burnett for homogeneous photopolymerization processes.^21^ Conversely, in 2004, Wan et al. reported a quantum yield increase for the formaldehyde formation over both bare and platinized TiO_2_. However, they obtained these results using pulsed laser illumination.^22^

Until now, there has been no evidence that CPI could work better than continuous illumination *at the same average photonic flow*, as demonstrated in many reprises.^18−20^

In two recent papers, Ardagh et al.^23,24^ theoretically demonstrated in an elegant formal manner that it is possible to enhance the rate of a catalyzed reaction through the decoupling of chemical-physical steps with different requirements by a square wave modulation of thermodynamic and kinetic-related properties of the couple catalyst/substrate. This effect, called catalyst surface resonance, occurs in a wide range of frequencies spanning more than 8 orders of magnitude, from 10 to 100 mHz up to 100 MHz,^23−25^ when the period of the applied surface modulation waveform is comparable with the characteristic times of the individual microkinetic reaction steps. In their last work, they observed this effect in the gas phase for the methanol reforming on Pt nanoparticles.^25^

In this work, taking inspiration from the work of Ardagh et al., we employed CPI to induce periodic variations in thermodynamic properties of a Pt/TiO_2_ system (e.g., reversible changes in the Fermi potential) to obtain, for the first time, an increase in the photonic efficiency *at the same average photonic flow*. To demonstrate our hypothesis, we carried out a Hydrogen Evolution Reaction (HER) on Pt/TiO_2_ slurries modulating the incident UV irradiation at three different frequencies (*f* = 1/*P*; 0.8, 80 Hz and 8 kHz), finding that the overall efficiency can be enhanced by more than 50% (for *f* = 80 Hz and 8 kHz) under CPI compared with continuous irradiation. Those frequencies were chosen because in our previous work we observed that irradiated TiO_2_ slurries start the transition from low- to high-frequency behavior around 0.1 Hz, while the surface catalytic resonance is maximized for frequencies from 2 to 5 orders of magnitude higher.

The photocatalytic experiments were carried out on bipyramidal TiO_2_ nanoparticles synthesized through a hydrothermal method (see Supporting Information (SI) Figure S1).^26^ The Pt nanoparticles were deposited through photoreduction under UV irradiation using formic acid as a hole scavenger (see SI Figure S2). The deposition time of the Pt onto the TiO_2_ bipyramids was fixed to 5 min under 20 W m^–2^ of continuous irradiation (details in SI). The experiments were done also on bare TiO_2_ in order to have a comparison and confirm the supposed mechanism.

To assess the influence of the CPI technique on the rate of H_2_ production, we perform three CPI experiments (*f* = 0.8 Hz, *f* = 80 Hz, and *f* = 8 kHz, with *I*^CPI^ = 100 W m^–2^ and γ = 0.2) and a continuous illumination experiment at an irradiance of *I*_0_ = 20 W m^–2^; in this way, we compared measurements with the same average incident irradiance over the entire irradiation experiment (see SI Figure S3).

Figure 2a highlights the strong effect of the CPI on the hydrogen evolution rates, with a more than 50% increase under 80 Hz and 8 kHz CPI (nearly 30% in the case of the 0.8 Hz). Conversely, without cocatalysts (Figure 2b), the CPI technique does not lead to an increase in the HER rate (i.e., the dark period is not contributing to the overall reaction as already observed).^18^ Pt-loaded materials produce H_2_ with greater efficiency (Figure 2c), coherently with the literature. CPI induces larger H_2_ production, with a significant increase from 0.8 to 80 Hz, as also witnessed by the more negative photopotential attained during CPI compared with continuous irradiation at the same average power (Figure 3a). The measured photopotentials at 0.8 and 80 Hz were 1.2 and 2.8 mV more negative than continuous, respectively. We observed a 3.2 mV potential shift at 8 kHz, in full accordance with the obtained photocatalytic hydrogen evolution rates. Confirmation of this evidence comes from photocurrent experiments (see SI Figures S6–S9), which further confirmed the photopotential trends. The photocurrents recorded are anodic and account for the electrons collected during the measurement. Those are the electrons not lost by recombination but also not transferred to the electrolyte to reduce protons to H_2_. Therefore, we expect photocurrents to have the same trend compared with photopotential, and, in fact, Pt-loaded materials have lower photocurrents compared with pristine TiO_2_, while photocurrents increase with periodic irradiation, paralleling the increased photopotentials observed with CPI.

**Figure 2:**
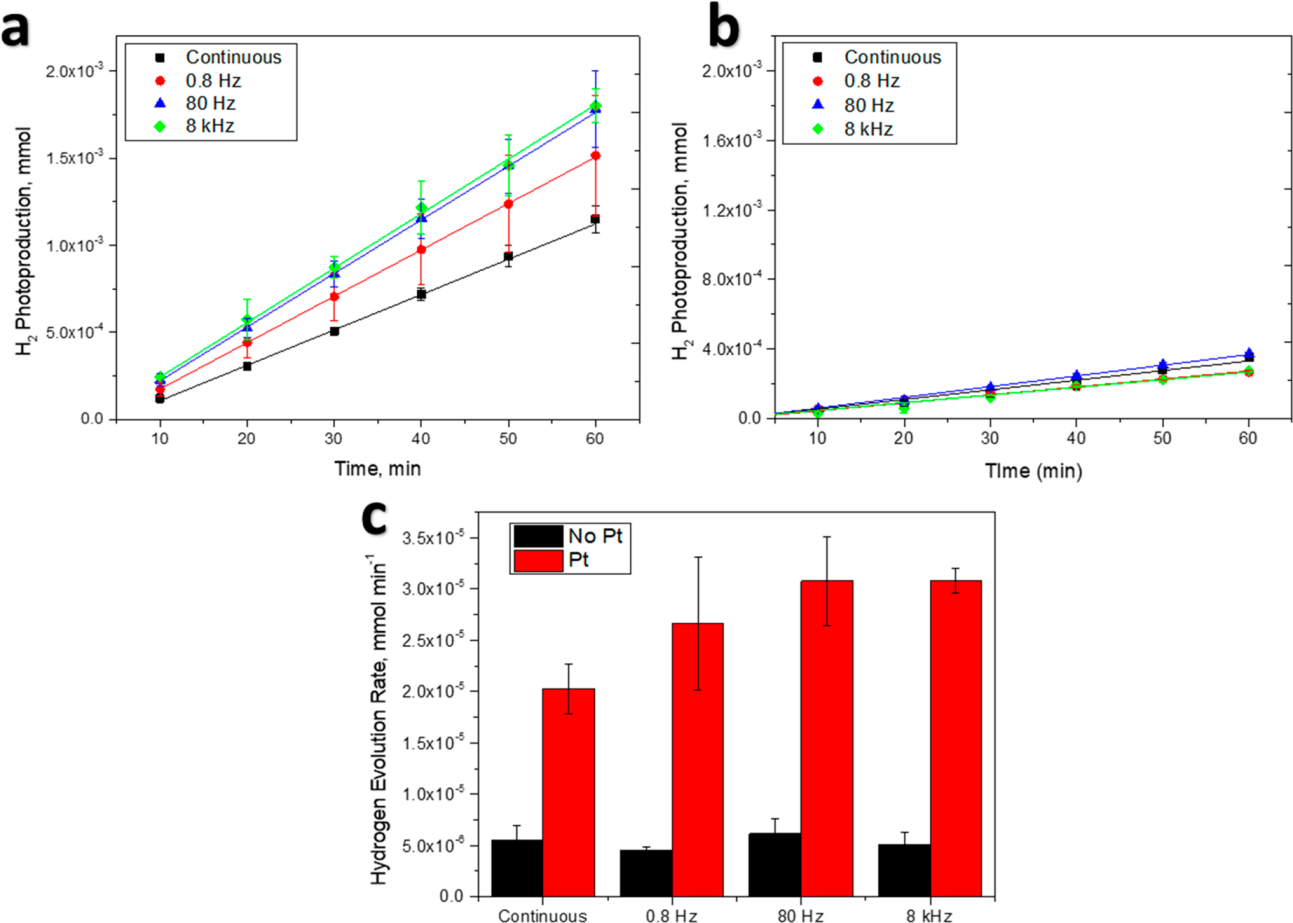
(a) H_2_ photoproduction during the 1 h tests over Pt-TiO_2_ nanoparticles; (b) H_2_ photoproduction during the 1 h tests over bare TiO_2_ nanoparticles; (c) H_2_ evolution rates over Pt-TiO_2_ and bare TiO_2_.

**Figure 3:**
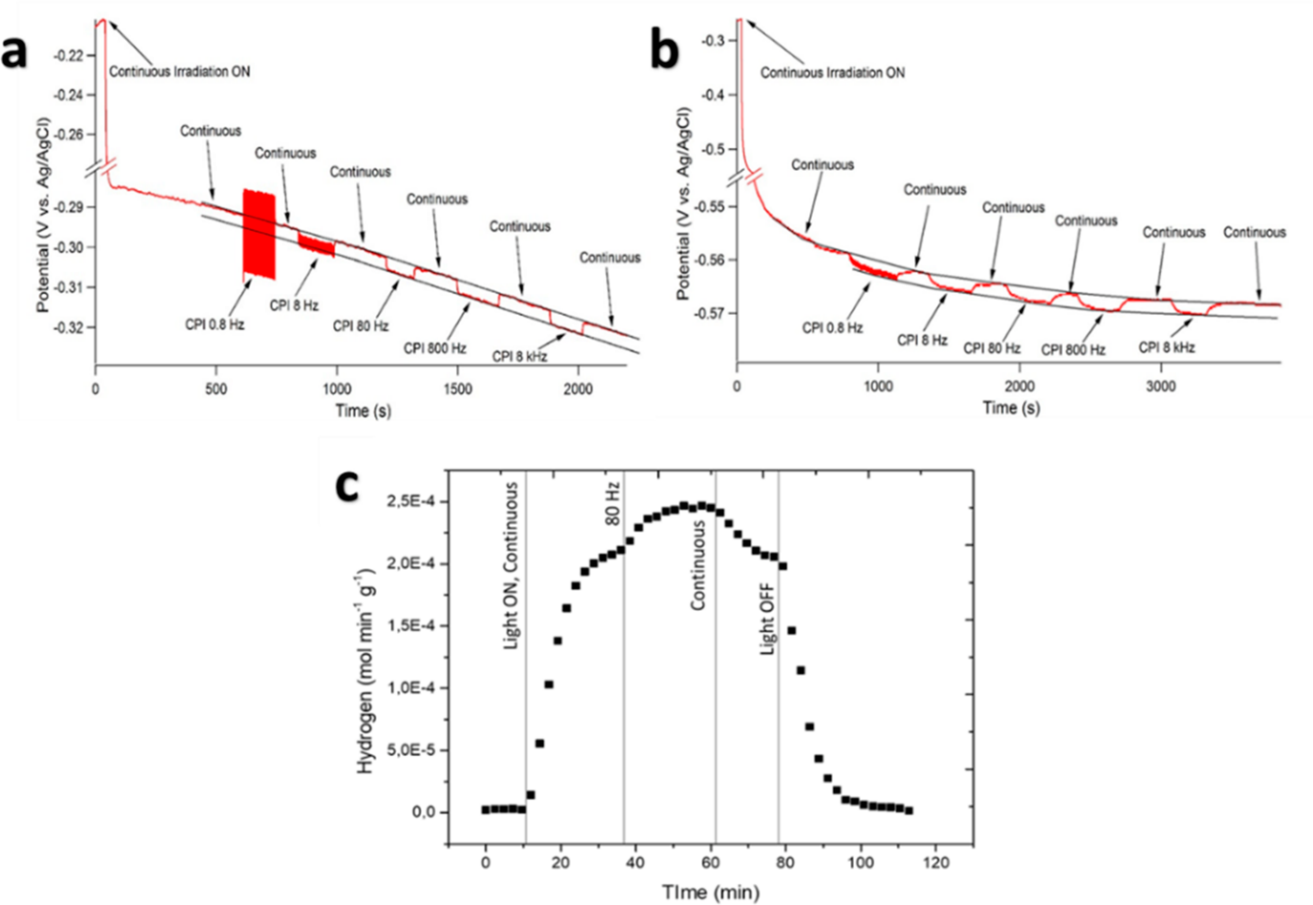
(a) Open Circuit Potential (OCP) measurements over a Pt-TiO_2_ electrode under different CPI conditions; (b) OCP measurements over a bare TiO_2_ electrode under different CPI conditions; (c) H_2_ production on the Pt-TiO_2_ electrode under irradiation without bias.

Therefore, during irradiation, each Pt island deposited on TiO_2_ works as a microelectrode at the potential imposed by TiO_2_. The small Tafel slope (30 mV)^27^ for HER on Pt allows faster H_2_ production under CPI. Such a small Tafel slope implies that a mere 3 mV shift in overpotential can be responsible for a 26% current increase, i.e., H_2_ evolution rate. We measured similar photopotential increases on the bare TiO_2_ nanoparticles (from 2.2 mV at 0.8 Hz, to 2.8 at 8 kHz, see Figure 3b). However, in the case of TiO_2_, the Tafel slope is significantly larger (more than 100 mV),^28,29^ and in this case, a 2 mV overpotential increase leads to less than a 4% increase in H_2_ evolution rate. Consequently, H_2_ production does not significantly improve with CPI. The improvement of the hydrogen evolution rate was confirmed also on the Pt-TiO_2_ electrode employed for the electrochemical analysis (Figure 3c). During this experiment, a 19% increase in H_2_ production at 80 Hz CPI compared to continuous irradiation at OCP was measured.

These results suggest that the explanation for the improvement is consistent with a different mechanism rather than nanoparticle deaggregation observed by Wang at al.^22^ In their report, they employed laser pulses to perform CPI, with a duty cycle of ∼10^–8^. To compare CPI with constant irradiation *I*_0_ with the same time-averaged power, they had set *I*_CPI_ ≈ 10^8^*I*_0_, whereas in our conditions *I*_CPI_ and *I*_0_ are in the same order of magnitude. Therefore, we cannot invoke a particle deaggregation mechanism caused by such an intense laser pulse to account for the improved H_2_ production rate under CPI.

The mechanism behind the improved photocatalytic activity on TiO_2_ supported metal nanoparticles has been extensively studied in the past.^30−32^ While the holes are scavenged by the sacrificial agent (HCOOH in this case) at the semiconductor surface, electrons are accumulated, leading to a negative shift of the Fermi potential.^14,33^ This potential displacement initiates H^+^ reduction on the Pt islands, which will be covered with Pt–H groups.

We observed that the Fermi potential becomes roughly 100 mV more negative during continuous irradiation (*I*_0_ = 20 W m^–2^). As evidenced in Figure 3a, even the lowest CPI frequency employed here (0.8 Hz) does not allow complete photopotential relaxation during the dark period. Nevertheless, we might hypothesize that the relaxation over the Pt surface is significantly faster than on the whole TiO_2_ particle (i.e., the only experimentally accessible), where electrons are trapped with slower transfer and detrapping rates. Consequently, if the Pt surface undergoes substantial potential changes under CPI conditions, then it will be possible to match the catalytic resonance constraints in which the H_2_ desorption is promoted during the dark interval (more positive potentials). An indirect proof of the different potential that can be can be reached by the Pt islands with respect to the TiO_2_ nanoparticles can be observed in Figure S10, where it can be observed that we were able to detect H_2_ even when the electrode was irradiated at +0.5 V vs Ag/AgCl (although the rate was reduced to 1/20 compared to OCP in Figure 3c). These findings confirm that the potential at the photodeposited Pt islands is significantly different from the electrode potential and during the CPI the potential at the Pt islands could fluctuate more than we can observe monitoring the OCP of the macroscopic electrode.

In conclusion, we demonstrated that the H_2_ evolution rate increases by more than 50% at frequencies higher than 80 Hz CPI compared with continuous irradiation with the same energy input. From a mechanistic point of view, surface catalytic resonance is a reasonable explanation of our observations, although we do not currently have conclusive evidence of the suggested hypothesis. We are presently working to confirm the surface resonance concept and generalize these encouraging results, varying duty cycle, irradiance, frequency, concentration of the hole scavenger, and the metallic cocatalyst (e.g., for the latter, we expect that the CPI could be even more effective for metals with large affinity for the proton, like W, Rh, and Ir). These significant findings open new scenarios to increase the quantum yield of the HER and, possibly, of the overall water photosplitting. Moreover, the CPI technique can be used as another valuable tool to unravel kinetic and thermodynamic features of photoinduced processes where surface catalysis is involved, e.g., artificial photosynthesis where a semiconducting photocatalyst is usually coupled with suitable cocatalysts to improve its reactivity.

## Supporting Information Available

Synthesis and characterization of the bipyramidal TiO_2_ nanoparticles, photocatalytic tests, and photoelectrochemical characterization (PDF)
