# Supplementary material for: Controlled Periodic Illumination Enhances Hydrogen Production by over 50% on Pt/TiO2
Source: ACS Catal. 2021 May 18;11(11):6484–8. doi: 10.1021/acscatal.1c01734 (PMC8294008; doi:10.1021/acscatal.1c01734)
Supplement: Supplementary file 1 — cs1c01734_si_001.pdf [file cs1c01734_si_001.pdf]

Supporting Information

Controlled Periodic Illumination Enhances Hydrogen Production by over 50% on Pt/TiO_2

F. Sordello, F. Pellegrino,* M. Prozzi, C. Minero, V. Maurino,*

[1]Dipartimento di Chimica and NIS Center, University of Torino, Via P. Giuria 7, 10125

Torino, Italy.

[2]JointLAB UniTo-ITT Automotive, Via Quarello 15/A, 10135 Torino, Italy.

Correspondence to: francesco.pellegrino@unito.it valter.maurino@unito.it

Materials and Methods

Synthesis of the TiO_2 nanoparticles

The synthesis of the bipyramidal nanoparticles was carried out by a hydrothermal method described in detail elsewhere.[1] Briefly, the precursor used for the hydrothermal synthesis is a complex of Ti(IV) with triethanolamine with molar ratio 1:2 ([Ti$(TeoaH)_2$]). The synthesis of the precursor is carried out as follows: in a three necked round bottomed flask 93 ml (0,7 mol) of triethanolamine (Aldrich Reagent grade 98%) is poured. The flask is equipped with a dropping funnel loaded with 104 ml (0,35 mol) of Ti(IV) isopropoxide (Aldrich reagent grade 98%). The flask and the funnels are maintained under a N_2 flow. The Ti(IV) isopropoxide is then dropped into the triethanolamine under vigorous stirring. A vacuum pump is attached to the flask, maintaining a gentle flow of N_2, until the isopropyl alcohol is distilled off. The synthesis product is a pale yellow viscous liquid, to be stored at 4°C under N_2 gas and used without further purification.

The synthetized complex is used as precursor for the hydrothermal synthesis at the following conditions:

- Precursor concentration: 65 mM;
- Triethanolamine: 40 mM;
- Initial pH: 10;
- Temperature: 220°C;
- Time: 50 hours

In general, after dissolution of the required mass of [Ti$(TeoaH)_2$] and (possibly) of the triethanolamine in ultrapure water (resistivity > 18.2 MΩ cm, TOC < 5 ppb, produced with a Millipore MilliQ apparatus), pH is adjusted with 0.1 M carbonate-free NaOH, as required. The

solution is then filtered through a 0.45 μm cellulose acetate membrane filter. The presence of iron in the precursor solution before hydrothermal treatment is checked by the thiocyanate test (Fe(III) < 0.2 mg L^{-1}). The solution is N_2 purged for at least 10 minutes in order to eliminate O_2 before sealing the hydrothermal reactor. The purging time should be adjusted in order to ensure an O_2 content of the gaseous phase in the reactor < 1% mol/mol. The reactor is heated to 40 °C for 30 minutes, then to the set temperature for the treatment (±1°C) at 1 °C min^{-1}. The temperature is kept constant for 50 hours. The reactor is then cooled in air.
At the end of the hydrothermal treatment the nanoparticles suspension is concentrated under vacum and then processed according to the following procedure:

1. Dialysis of the concentrated raw suspension against ultrapure water (MilliQ, Millipore) using a Spectra/Por dialysis membrane tubing (MWCO 8-12 kD or MWCO 12-14 kD), final pH of the permeate in the range 5-8, Cl^- and SO_4^{2-} < 1 mg L^{-1} (by ion chromatography).
2. Freeze-drying of the suspension.
3. Resuspension in milli-Q water (ca. 100 g/L)
4. Addition of 5 g L^{-1} of H_2O_2 and irradiation (in air) for 24 hours of the suspension, after addition of 50 g/L H_2O_2, under UV light using a Medium Pressure Mercury Lamp (emission wavelength 360 nm) or a fluorescent blacklit lamp (360 nm), ca. 20 W/m^2 in the range 300-400 nm.
5. Dialysis against milli-Q water using a Spectra/Por dialysis membrane tubing (MWCO 8-12 kD or MWCO 12-14 kD), final pH of the permeate in the range 5-8, Cl^- and SO_4^{2-} < 1 mg L^{-1} (by ion chromatography).
6. Freeze-drying of the suspension.

Finally, the powders obtained was calcined at 600°C in air for one hour in order to eliminate any presence of residual organic molecules from the surface (Figure 1).

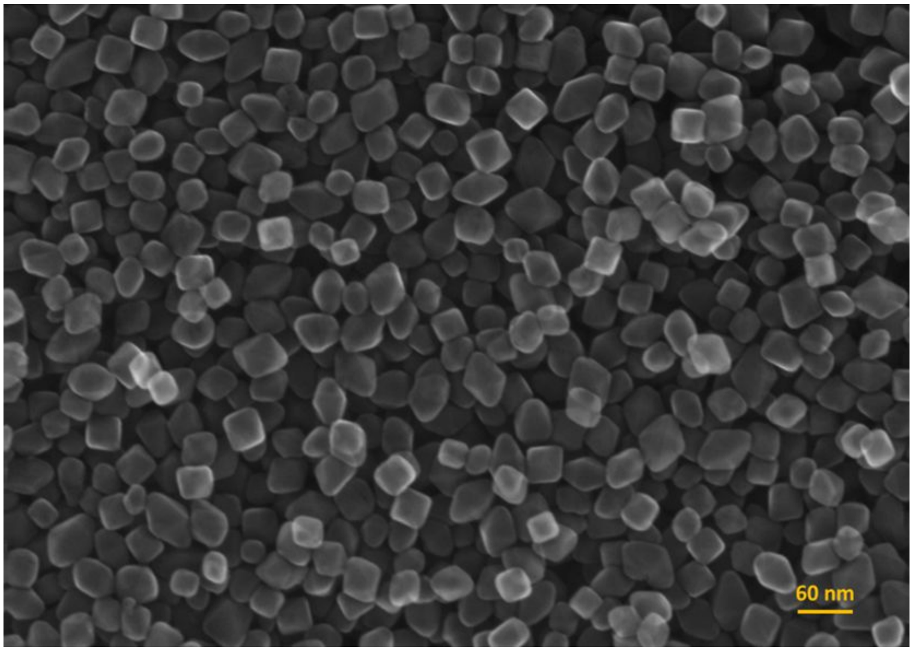

Figure S1. Scanning Electron Microscopy (SEM) micrograph of the bipyramidal TiO_2 nanoparticles used as photoactive material.

Photocatalytic Tests

Hydrogen production experiments were performed irradiating with UV light slurries containing 1000 mg L^{-1} of TiO_2 powder of the materials and 20 mg L^{-1} of Pt, added as H_2PtCl_6, which is reduced to Pt^0 (by the photoelectrons produced in the semiconductor) and deposited onto TiO_2 upon irradiation (Figure S2). TEM micrographs on a similar system was already carried out in a previous paper.[2] The suspension used has been always the same in order to avoid differences between the experiments: 100 ml of TiO_2 bipyramids 1 g L^{-1} with 0.5 M of formic acid, pH 1.6. The irradiation experiments were carried out in magnetically stirred, cylindrical quartz cells (3.5 cm inner diameter, 2 cm height), where 5 mL of slurry (5 mg of material) are introduced together with 1 ml of H_2PtCl_6. The deposition of the Pt was maintained constant for each experiments: 5 min under continuous irradiation at 20 W m^{-2}. Before irradiation the cell containing the slurry was carefully purged with nitrogen to remove oxygen from the reaction environment (5 min at 100 ml/min). The removal of oxygen is fundamental in order to avoid the competitive oxygen reduction reaction. The procedure for the experiments carried out without co-catalyst is the same but no H_2PtCl_6 was added before the irradiation. Each experiment was triply repeated in three different days, each time randomizing the order of execution.

The irradiation was carried out with a LED source centered at 365 nm. The irradiation system is fully described in a previous our paper.[3] The waveforms employed are shown in Figure S3, Figure S4, Figure S5 and in Table S1 in more details.

Hydrogen evolution was followed sampling periodically 2.5 mL of gas from the irradiation cell and replacing it with the same volume of N_2. The gas sample was analyzed with an Agilent 490 Micro GC gas chromatograph equipped with a Molsieve 5Å column for H_2 analysis. During the analysis the column were kept at temperatures of 363 K at a pressure of 200 kPa. The carrier gas was argon. The total amounts of H_2 produced as function of time were calculated from the

concentration in the sampled gas, considering the total volume of gas in the irradiation cell and the previous samplings.

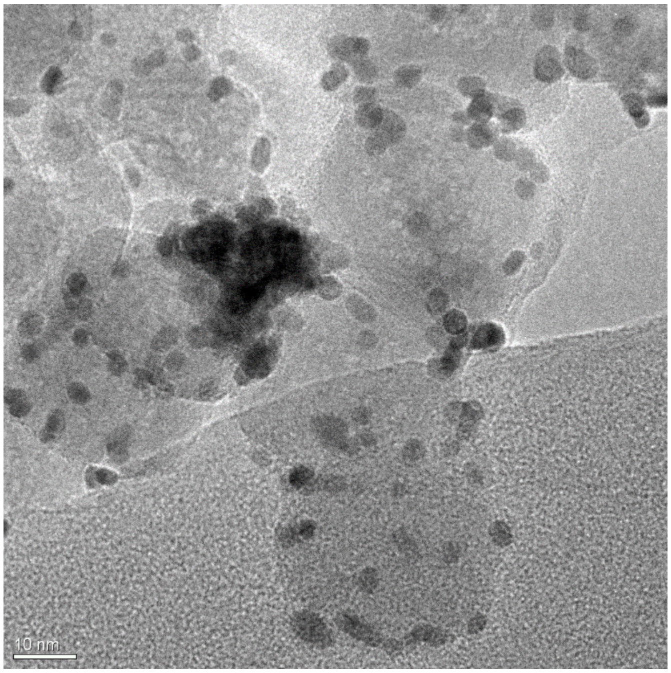

Figure S2. TEM analysis of the Pt nanoparticles deposited onto the TiO_2 bipyramids

Photoelectrochemical Characterization

The photoelectrochemical characterization was carried out on TiO_2 electrodes produced by dropping 1 ml of the TiO_2 or Pt-TiO_2 suspension over a fluorine doped tin oxide (FTO) glass drying in air at room temperature. The illuminated area of the electrodes is 4 cm^{-2}.

The electrochemical measurements were performed using a standard photo-electrochemical setup, composed of a computer-controlled potentiostat, AUTOLAB PGSTAT12, and the same light source employed for the H_2 evolution experiments. The electrochemical cell was a conventional three-electrode cell with a 1 mm thick fused silica window. The counter and reference electrodes were a Glassy Carbon and a Ag/AgCl/KCl (3M) electrode, respectively. Both CV and OCP measurements were carried out under a N_2 atmosphere (flux 200 mL min-1); the solution (0.5 M formic acid) was purged with N_2 for 30 min before each measurement in order to eliminate the residual O_2 present in the solution, to obtain the recombination rate of the photogenerated carriers. The experiments are carried out at at pH 1.6 to reproduce the same experimental conditions adopted during photocatalytic hydrogen evolution. Figure S6 shows the photoelectrochemical measurements on the bare TiO_2.

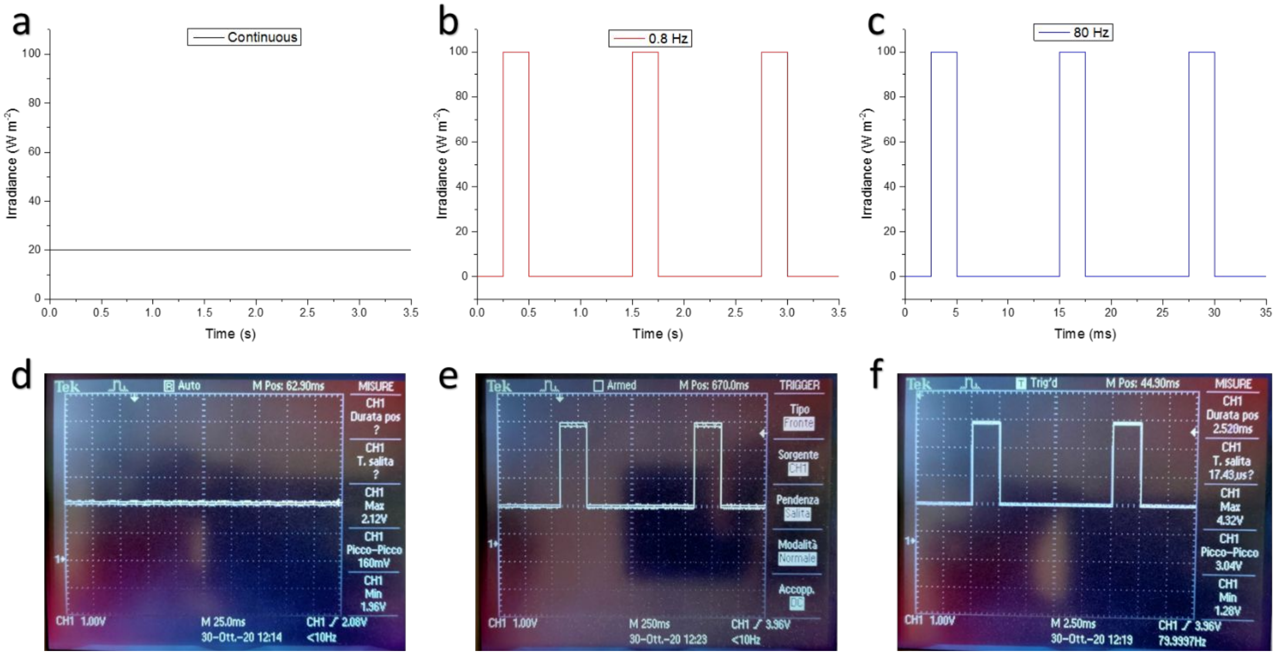

Figure S3. Nominal irradiation profile of the three different experiments carried out in this work together with the waveforms profiles used to drive the LEDs: a) and d) continuous; b) and e) 0.8 Hz; c) and f) 80 Hz

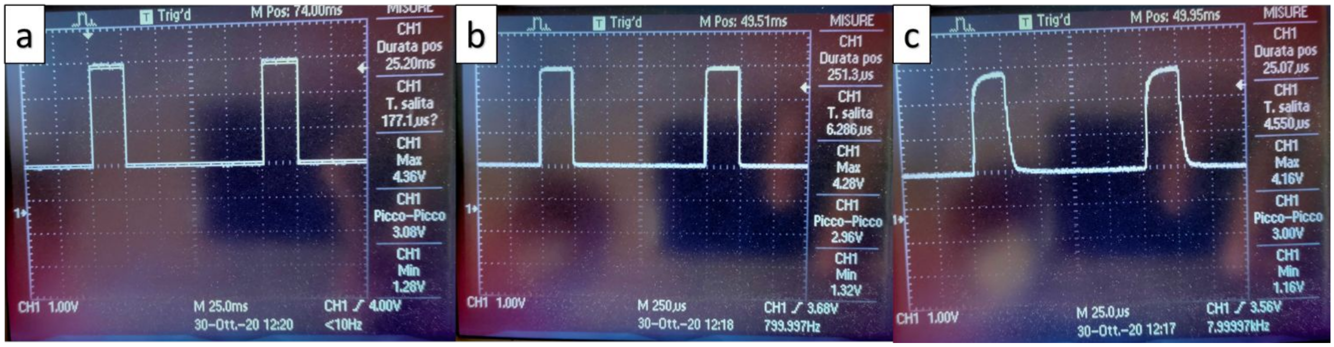

Figure S4. Light pulses generated by the LEDs with the drive waveforms reported in Figure S2 and detected with a photodiode: a) 8 Hz; b) 800 Hz; c) 8kHz (see ref. 2 for details on the measurements)

We verified that the illumination system is able to provide square wave light pulses (for the various frequencies employed in this study) in which:

- during the light pulse the irradiance value is actually the one chosen (i.e. $I^{CPI} = I_0 \cdot \gamma^{-1} = 100\ W\ m^{-2}$);
- during the dark time the irradiance value is 0.

The average voltage value recorded by the oscilloscope when the photodiode is exposed to the dark and to continuous illumination at an irradiance of 100 W m^{-2} are respectively: $\mathbf{V_{Dark} = 1.36\ V}$ and $\mathbf{V_{Continuous} = 4.24\ V}$. The following table (Table S1) reported the highest and the lowest voltage value recorded by the oscilloscope when $I^{CPI} = 100\ W\ m^{-2}$ and $\gamma = 0.2$ for the five different frequencies employed in the work.

Frequency (Hz)	MAX Voltage (V)	MIN Voltage (V)
8000	4.2	1.16
800	4.28	1.28
80	4.28	1.28
8	4.32	1.28
0.8	4.32	1.28

Table S1. Maximum and minimum voltages obtained at different frequencies employed

Hence, the reaction cell is effectively irradiated at $I^{CPI} = I_0 \cdot \gamma^{-1}$ during the light pulse. Considering that the voltage value recorded during CPI experiments are slightly lower than the one recorded under dark exposure of the photodiode, it is possible to state that for the dark time the effective irradiance of the LED source is equal to 0. We do not employed frequency higher than 8 kHz because our lighting system is not able to provide light pulses with a rise and decay times less than 10% of the pulse duration.
The figure S4 shows the light pulse profile for a drive waveform with f = 8 kHz:

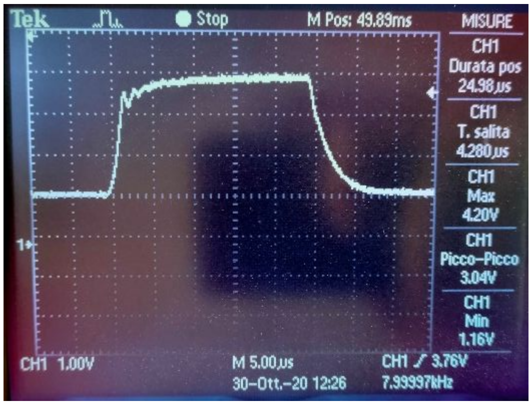

Figure S 5. Light Pulse profile obtained with the drive waveform at 8 kHz

In this case, we observed a rise and a down time of, respectively, 4.3 and 5.3 µs. Therefore, the shape of the square wave is subjected to a variation of about 32 % of the light time with a consistent distortion of the ideal square waveform. Anyway, for the other frequencies, these values for rise and decay time are negligible when compared with the entire duration of the pulse (> 250 µs).

The enhancement of the photocatalytic activity was evaluated by performing chronoamperometry experiments on both the TiO_2 and Pt-TiO_2 electrodes at two different imposed potentials: +0.5 V (Figure S6) and -0.2 V (Figure S8). The choice of these two potentials was made to mimic the conditions during the irradiation of the slurries in the case of -0.2 V, and to work sufficiently far from the flat band to minimize the dark currents in the case of 0.5 V. Nevertheless the results at the two different potential values were similar, and in both cases, the photocurrents ($I_{LightON}$ - $I_{LightOFF}$) increased under CPI (only for 0.8 Hz at -0.2V does the current seem to be lower, however at 0.8 Hz the measurements are intrinsically less accurate, due

to the large oscillations of the current during irradiation). The $I_{LightON}$ and $I_{LightOFF}$ values were calculated by averaging (for 10 s at least) the currents in dark and under illumination. For bare TiO_2, the increase is the same at all the frequencies (even higher at 0.8 Hz, but with larger uncertainty), while in the case of the Pt-TiO_2 electrode, the increment is higher at 80 Hz and 8 kHz (see Figure S7 and Figure S9).

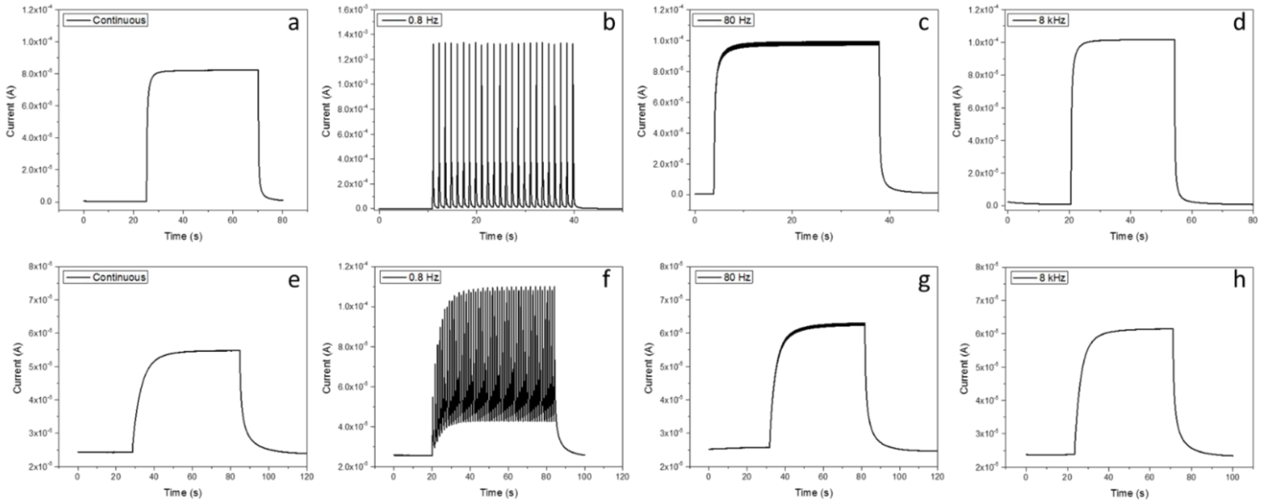

Figure S6. Chronoamperometry tests at +0.5V (vs Ag/AgCl) for the TiO_2 (a, b, c, d) and Pt-TiO_2 (e, f, g, h) electrodes.

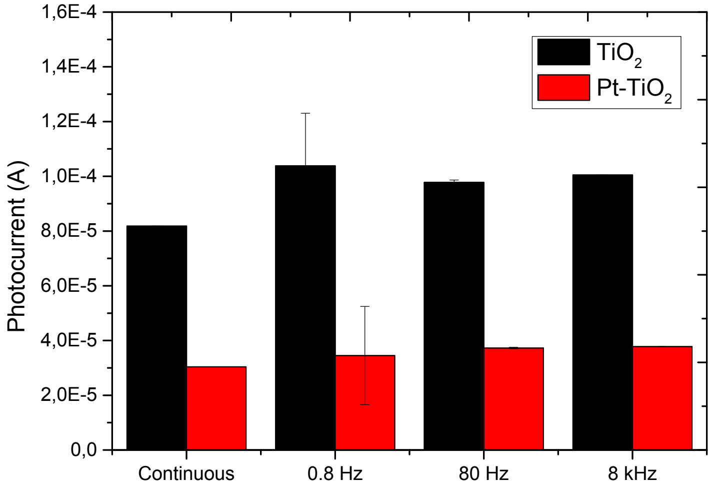

Figure S7. Photocurrent obtained from the chronoamperometry experiments carried out at +0.5V.

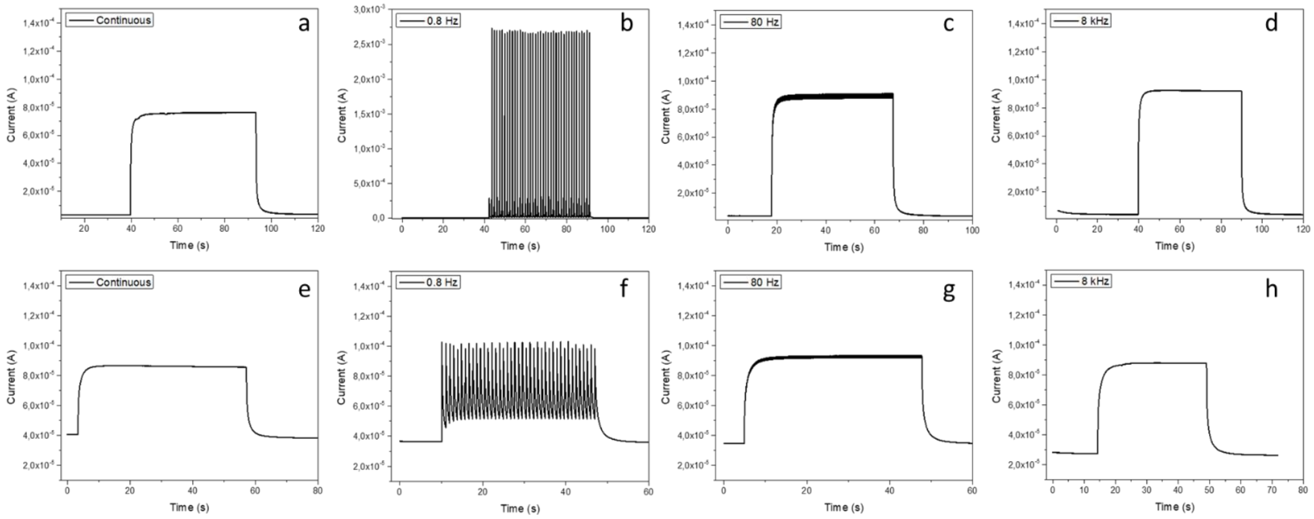

Figure S8. Chronoamperometry tests at -0.2V (vs Ag/AgCl) for the TiO_2 (a, b, c, d) and Pt-TiO_2 (e, f, g, h) electrodes.

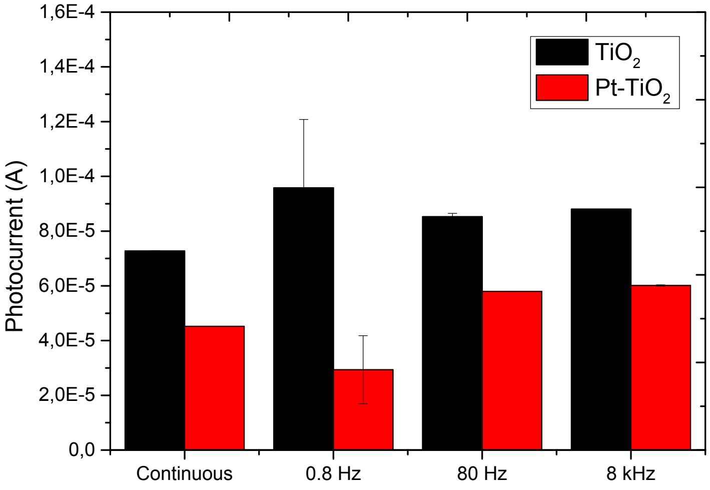

Figure S9. Photocurrent obtained from the chronoamperometry experiments carried out at -0.2V.

The lower photocurrent observed for the Pt-loaded material is perfectly coherent with the faster H_2 production kinetics, and also with the lower photopotential sustained by the Pt-loaded sample. It must be underlined that we are not in presence of an inert electrolyte. In that case, charge carriers would be mainly lost by recombination, and the material with the slowest recombination kinetics would display the larger photocurrents. HCOOH is an efficient hole scavenger, inihibits recombination and leaves photoelectrons in the electrode. Pt islands provokes H_2 reduction consuming electrons that no longer contribute to the photocurrent, thereby reducing photocurrents.

Given the results obtained for the photocurrents, we measured the H_2 production under bias. This measurement has been useful to understand the quasi Femi level dynamics inside the Pt-loaded material. The electrochemical cell was coupled to a gas-chromatograph and the H_2 produced under irradiation was measured at two different conditions: at OCP and with a +0.5 V vs Ag/AgCl bias with the Pt-TiO_2 electrode immersed in a 0.5 M HCOOH electrolyte. During these experiments, 19 % increase in H_2 production at 80 Hz CPI compared to continuous irradiation at OCP was measured (Figure 3c). We were also able to detect H_2 even when the electrode was irradiated at +0.5 V vs Ag/AgCl, although the rate was reduced to 1/20 compared to OCP (Figure S10).

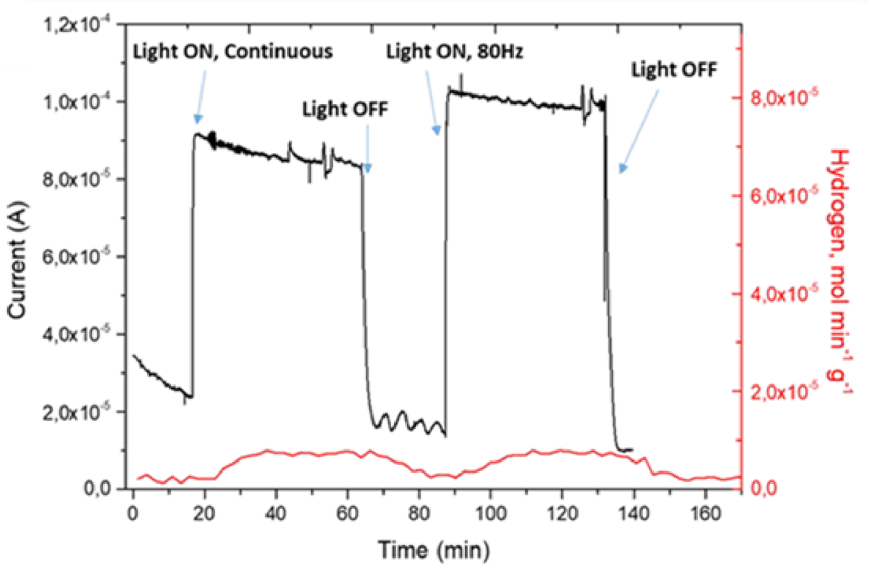

Figure S10. H_2 production under +0.5 V (vs Ag/AgCl) bias.

References

1. Pellegrino, F.; Isopescu, R.; Pellutiè, L.; Sordello, F.; Rossi, A. M.; Ortel, E.; Martra, G.; Hodoroaba, V.-D.; Maurino, V., Machine Learning Approach for Elucidating and Predicting the Role of Synthesis Parameters on the Shape and Size of Tio2 Nanoparticles, *Scientific Reports* **2020,** 10 (1).
2. Pellegrino, F.; Sordello, F.; Mino, L.; Minero, C.; Hodoroaba, V.-D.; Martra, G.; Maurino, V., Formic Acid Photoreforming for Hydrogen Production on Shape-Controlled Anatase Tio2 Nanoparticles: Assessment of the Role of Fluorides, {101}/{001} Surfaces Ratio, and Platinization, *ACS Catalysis* **2019,** 9 (8), 6692-6697.
3. Prozzi, M.; Sordello, F.; Barletta, S.; Zangirolami, M.; Pellegrino, F.; Bianco Prevot, A.; Maurino, V., Assessing a Photocatalytic Activity Index for Tio2 Colloids by Controlled Periodic Illumination, *ACS Catalysis* **2020,** 10 (16), 9612-9623.
